# Effects of Three-Dimensional Circular Truncated Cone Microstructures on the Performance of Flexible Pressure Sensors

**DOI:** 10.3390/ma15134708

**Published:** 2022-07-05

**Authors:** Weikan Jin, Zhiheng Yu, Guohong Hu, Hui Zhang, Fengli Huang, Jinmei Gu

**Affiliations:** 1 School of Mechanical Engineering and Automation, Zhejiang Sci-Tech University, Hangzhou 310018, China; jinweikan@163.com; 2 Key Laboratory of Advanced Manufacturing Technology of Jiaxing City, Jiaxing University, Jiaxing 341000, China; 2112002281@zjut.edu.cn (G.H.); jmgu1218@zjxu.edu.cn (J.G.); 3 College of Mechanical and Electrical Engineering, Jiaxing Nanhu University, Jiaxing 314000, China; yuzhiheng@jxnhu.edu.cn

**Keywords:** microstructures, low-frequency ultrasonic resonance printing, flexible pressure sensor, sensitivity, response time

## Abstract

Three-dimensional microstructures play a key role in the fabrication of flexible electronic products. However, the development of flexible electronics is limited in further applications due to low positioning accuracy, the complex process, and low production efficiency. In this study, a novel method for fabricating three-dimensional circular truncated cone microstructures via low-frequency ultrasonic resonance printing is proposed. Simultaneously, to simplify the manufacturing process of flexible sensors, the microstructure and printed interdigital electrodes were fabricated into an integrated structure, and a flexible pressure sensor with microstructures was fabricated. Additionally, the effects of flexible pressure sensors with and without microstructures on performance were studied. The results show that the overall performance of the designed sensor with microstructures could be effectively improved by 69%. Moreover, the sensitivity of the flexible pressure sensor with microstructures was 0.042 kPa^−1^ in the working range of pressure from 2.5 to 10 kPa, and the sensitivity was as low as 0.013 kPa^−1^ within the pressure range of 10 to 30 kPa. Meanwhile, the sensor showed a fast response time, which was 112 ms. The stability remained good after the 100 cycles of testing. The performance was better than that of the flexible sensor fabricated by the traditional inverted mold method. This lays a foundation for the development of flexible electronic technology in the future.

## 1. Introduction

Flexible electronic devices play an increasingly significant role in devices and systems of electronic skin [1,2,3], human–computer interactions [4], and physiological signal monitoring [5]. Flexible pressure sensors are an important part of flexible electronic devices, which can sense a small range of pressure, and convert it into electrical signals [6]. The sensing principles of flexible pressure sensors include capacitive sensing [7], piezoresistive sensing [8], piezoelectric sensing [9], and triboelectric sensing [10]. Flexible pressure sensors manufactured by different sensing principles have different application scenarios and characteristics [11]. At present, flexible pressure sensors have become a research hotspot because of their simple structure, simple tests, low preparation cost, and high sensitivity [12].

To improve the linear range and sensitivity of flexible pressure sensors, research into the microstructure has been the main research direction. Flexible pressure sensors with a micro-column structure and a pyramid structure are common geometric structures. Zhang [13] prepared a flexible pressure sensor with a height-adjustable micro-column array. This sensor had a high shear force sensitivity (4.48 kPa^−1^) and could accurately measure the shear force. Sally [14] et al. designed and developed an innovative and sensitive flexible sensor for efficient potentiometric monitoring of Ni (II) ions, which was constructed from highly porous activated flexible carbon cloth decorated with nitrogen and spherical porous carbon nanoparticles derived from low-cost cotton doped with polypyrrole nanoparticles via a simple carbonization–activation process followed by dip-coating in a membrane cocktail containing 2D Ni-MOF nanosheets as an electroactive material. The detection limit of the sensor was 2.7 × 10^−6^ with the pH range of 2–8. Nour [15] et al. developed a method of synthesizing polyaniline–polypyrrole composite materials with a network morphology, and prepared a thin layer of polypyrrole. Sally [16] et al. prepared a nanocomposite, and developed new sensitive and selective modified carbon paste electrodes (CPE), which showed good discriminating capability toward ClO-HCl with regard to a number of interfering materials. Choi [17] prepared PDMS films with pyramid microstructures, and spin-coated conductive active materials on their surfaces. The flexible pressure sensor manufactured by this method had the advantages of a large measurement range (10–100 kPa) and a fast response time (210 ms). To improve the performance of the sensor elements, microstructures such as spherical microstructures [18], bionic microstructures [19], folded microstructures [20], porous microstructures [8], and so on, are also being studied. Therefore, a reasonably designed microstructure is an effective method to fabricate highly sensitive flexible pressure sensors. Most of the research on the microstructure of flexible pressure sensors has mainly focused on the active material layer, and little research has focused on the fabrication of electrode layer microstructures. To facilitate their application, the manufacturing of flexible pressure sensors must have the characteristics of miniaturization and array. The preparation methods of the microstructure have the characteristics of diversity and comprehensiveness because of the complexity of the materials. Sun [21] coated a conductive polymer on a sandpaper template, obtaining a layered spinel microstructure. Ji [22] placed the cured polymer in water to dissolve NaCl to prepare an elastomer with a porous structure by using the template etching method. Chai [23] added Ca^2+^ as crosslinking agent to a graphene oxide sol to convert it into printable gel ink, and prepared a porous conductive network structure by using 3D printing technology. Because these methods need many processing steps and a complex operation, it is difficult to realize large-area repetitive manufacturing of microstructures without an efficient production process.

In view of the existing problems, in this work, we propose a new low-frequency ultrasonic resonant printing technology for high-precision and high-efficiency fabrication of microstructures. A flexible pressure sensor with microstructured electrodes was designed by using low-frequency ultrasonic resonant printing technology. The fabrication method of the sensor is introduced in this study. The surface structure of each part of the sensor was characterized, and the related tests were carried out after packaging. The effects of the microstructured electrode on the performance of the sensor were compared. The test results showed that the electrode with a microstructure could effectively improve the performance of the sensor, and the sensor had a fast response time and good repetition stability. It has great application potential in the field of flexible electronic devices.

## 2. Materials and Methods

### 2.1. Materials

Multi-walled carbon nanotubes (MWCNTS, outer diameter, 110~190 nm; length, 5~9 μm; Chengdu Zhongke Times Nano Energy Tech Co., Ltd., Chengdu, China), polydimethylsiloxane (PDMS, Sylgard 184, Nanjing Danpei Chemical Co., Ltd., Nanjing, China), isopropyl alcohol (IPA, Shanghai Macklin Biochemical Co., Ltd., Shanghai, China), silicone precision film (Model BD film KRR-200, Hangzhou Guizhi New Material Technology Co., Ltd., Hangzhou, China), silver nanoparticle ink (solid content, 20~30 wt%; 10~15 cp; 30 nm; Xi’an Qiyue Biotechnology Co., Ltd., Xi’an, China).

### 2.2. Structure and Printing Mechanism of the Flexible Pressure Sensor

**Figure 1:**
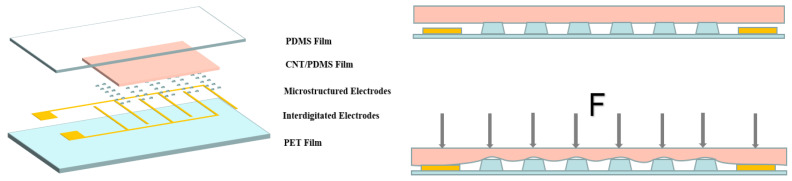
Diagram of the structure and working principles of the flexible pressure sensor.

The bottom layer of the flexible pressure sensor was a layer of flexible PET film. The top layer of the flexible PET film was an electrode, which was composed of an interdigital electrode and microstructures. To ensure the sensor had high sensitivity, the flexible film for the uppermost package of the flexible pressure sensor was made of PDMS material, which had good elasticity. The lower layer of the PDMS film was a rough active material layer prepared from CNT/PDMS composites, which had excellent electrical conductivity. CNT/PDMS composites are widely used in pressure sensors. A schematic diagram of the structure and working principles of the sensor is shown in Figure 1.

In the initial state without external pressure, the sensor had the highest contact resistance, because the surface of the active material layer was only in contact with the microstructured electrodes, the contact area was minimal, and the active material layer was not deformed. Under the application of external pressure, compression of the active material layer led to the complete connection of adjacent CNTs in the rough CNT/PDMS film to form a conductive network, which increased the electrical conductivity of the composite. Secondly, after the external pressure was applied, the active material layer gradually contacted the interdigitated electrodes, resulting in an increase in the contact area to increase the conductive path and reduce the resistance. The electrode with a microstructure improved the performance of the sensor in two aspects. Firstly, the active material layer and the interdigital electrode were separated without an external force, which increased the initial resistance and improved the sensitivity of the sensor. Secondly, the microstructured electrodes allowed the active material layer to deform more when pressure was applied; thereby, the performance of the sensor was improved.

## 3. Fabrication of Flexible Pressure Sensors

### 3.1. Fabrication of Microstructures

**Figure 2:**
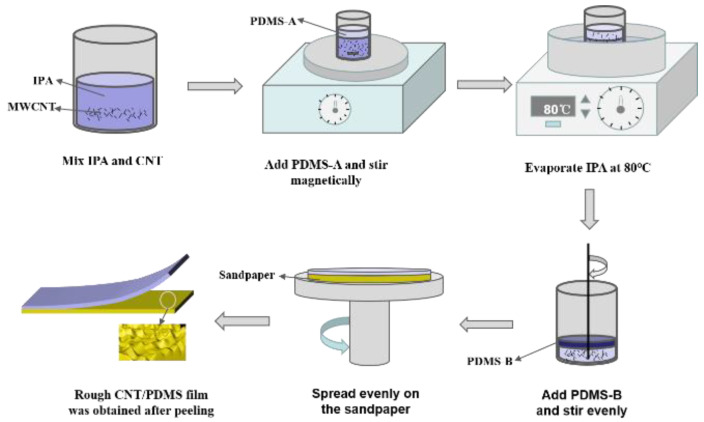
Flowchart of CNT/PDMS film preparation.

The preparation of the flexible pressure sensor mainly included the preparation of the rough CNT/PDMS composite active material layer and the preparation of the electrode layer with microstructures. The rough CNT/PDMS composite active material layer was made of PDMS and MWCNT. Sandpaper was used as a template. This material was prepared by doping MWCNT with PDMS. It was prepared by ultrasonication, coating, curing, stripping, and other processes. The preparation process is shown in Figure 2.

The detailed preparation process was as follows. Firstly, MWCNT and isopropanol were mixed at a weight ratio of 50:1, and ultrasonicated for 1 h. Secondly, the solution was added to PDMS-A, and the solution was placed on a magnetic mixer to stir evenly for 4 h to ensure the MWCNT and PDMS were fully mixed. Thirdly, the solution was heated in an oil bath. It was stirred at 80 °C for 2 h to fully volatilize the isopropanol in the solution. Fourthly, a curing agent (PDMS-B) was added and stirred evenly. Then the mixture was put into a vacuum drying oven and dried at room temperature for 1 h to remove bubbles, then CNT/PDMS composites were prepared. Fifthly, the mixture was uniformly coated onto sandpaper by a rotary coater. It was then cured in an oven at 60 °C for 2 h. Finally, it was stripped to obtain a rough CNT/PDMS film.

**Figure 3:**
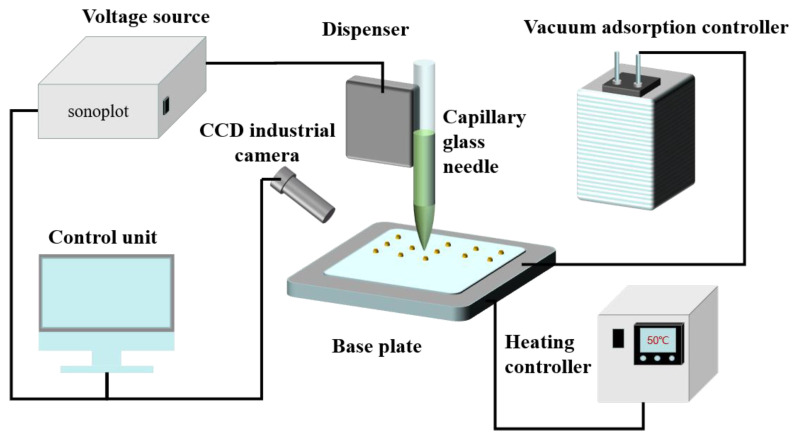
Structural diagram of the printing equipment.

The microstructure array of the electrode layer and the interdigital electrode were prepared by SonoPlot GIX Microplotter II printing equipment. The equipment was mainly composed of a high-precision mobile operation platform, a real-time monitoring CCD industrial camera, a capillary glass needle, a distributor, a voltage source, a vacuum adsorption controller, a heating controller, and a control unit. The equipment is shown in Figure 3. Its working principle was that the liquid at the tip of the glass tube needle contacted the surface of the substrate to form a “liquid bridge” to connect with the substrate. At the same time, the distribution voltage was applied to make the glass tube vibrate, which ensured the continuous flow of liquid from the tip.

**Figure 4:**
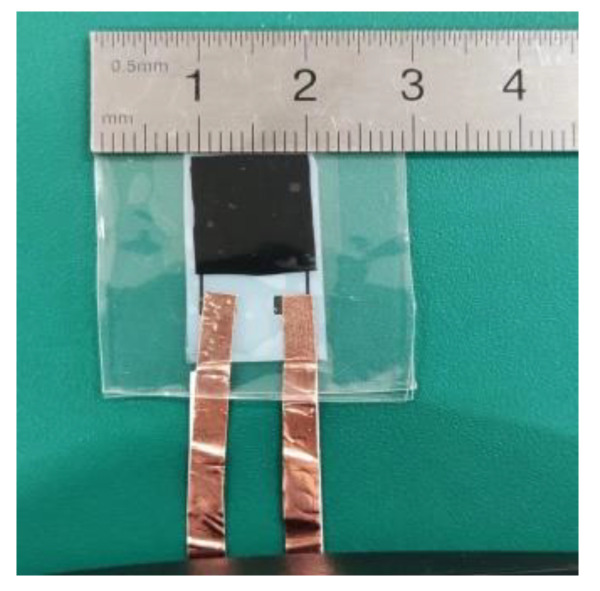
Physical layout of the electrode layer.

The preparation process was as follows. Firstly, the configured silver nanoparticle ink was sucked into the capillary needle tube by the capillary effect. Secondly, the vacuum adsorption device was opened to adsorb the PET substrate on the platform. Thirdly, the capillary needle tube was slowly brought close to the PET substrate by the control unit, so that the liquid at its tip contacted the substrate surface to form a liquid bridge, which connected to the substrate. The system controlled the output distribution voltage of the voltage source to make the needle tube vibrate, and then the control unit controlled the movement of the capillary needle to carry out the patterning process. Finally, the sample was placed into a vacuum drying oven and dried under a vacuum at 60 °C for 12 h to complete the fabrication of the electrode layer. The prepared sample is shown in Figure 4.

### 3.2. Morphological Characterization of the Microstructures

**Figure 5:**
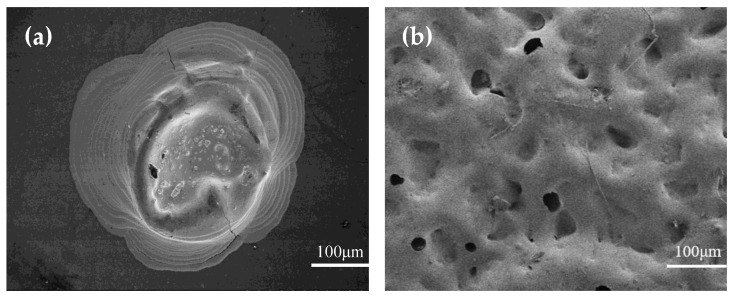
SEM images of the microstructure and CNT/PDMS film. (**a**) Surface morphology of the microstructure (**b**) Surface morphology of the thin CNT/PDMS films.

To study the surface morphology of the prepared microstructures and the active material layer, the microstructure’s surface morphology was characterized by emission scanning electron microscopy, and the results are shown in Figure 5.

As seen in Figure 5a, microstructures could be printed by the printing equipment, which was layer-by-layer printing. The ink did not appear to escape when the microstructure was being printed, because one thin film of silver nanoparticle ink was deposited in a picoliter volume, so the film dried in a very short time at room temperature.

In Figure 5b, it can be seen that the surface microstructures of the CNT/PDMS films prepared by sandpaper had irregular pore structures. However, the rough surface could improve the sensitivity of the flexible pressure sensors, because it could deform more easily than a smooth one under the same pressure.

## 4. Results

### 4.1. The Effects of Microstructures on Flexible Pressure Sensors’ Sensitivity

**Figure 6:**
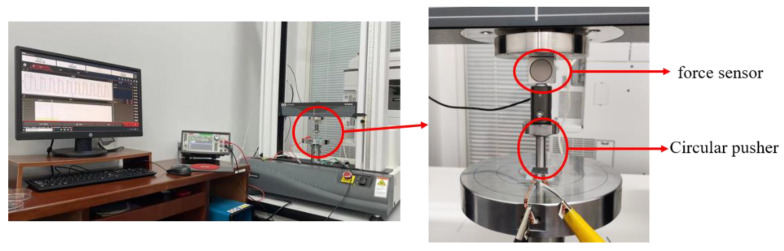
Sensitivity testing device used for the sensor.

To evaluate the sensing performance of the sensor, the flexible pressure sensor was connected to the electrical sensing analyzer through conductive copper wires, and it was placed on the pressure testing machine for real-time sensing recording. The testing device is shown in Figure 6. The electronic universal material testing machine set the force loading mode on the computer, and monitored the applied force in real time through the force sensor. External force was applied to the sensor through a circular loader with a diameter of 10 mm.

**Figure 7:**
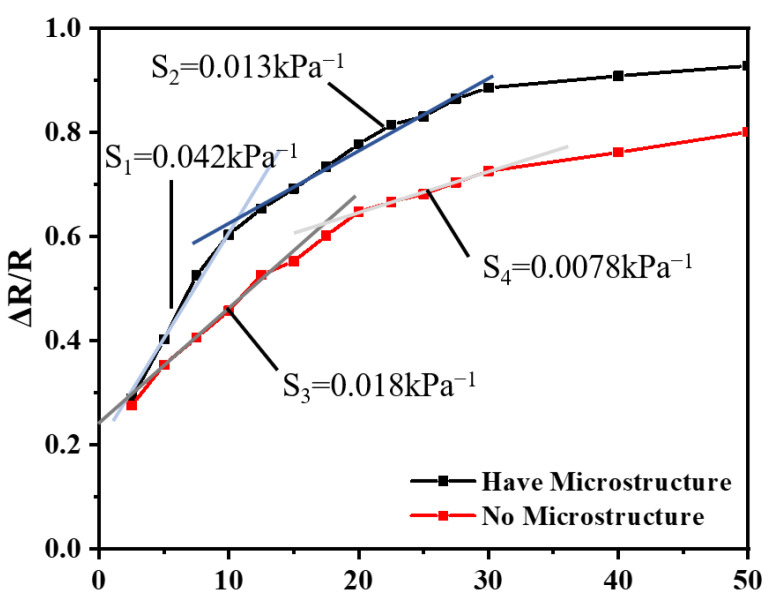
Relationship between the rate of change in the resistance and pressure of sensors with and without microstructured electrodes.

To study the effects of the electrode with microstructures on the sensor, two flexible pressure sensors with and without microstructured electrodes were prepared and tested. The formula for calculating sensitivity (S) is as follows:

S = [(R − R_0_)/R_0_]/Δp (1)

where R_0_ is the initial resistance of the device without pressure, R is the resistance after applying pressure, and Δp is the change in the pressure value. The sensitivity of the flexible pressure sensor with and without microstructures under different pressures is shown in Figure 7. It was demonstrated that the sensitivity of the flexible pressure sensor with microstructures was significantly better than that of the one without microstructures. The sensitivity with microstructures was 0.042 kPa^−1^ under pressure from 2.5 to 10 kPa, and the sensitivity was 0.013 kPa^−1^ under pressure from 10 to 30 kPa, which was lower by more than 69%. The performance was better than that of the flexible sensor fabricated by the traditional inverted mold method. This was due to the difference in the contact area when the sensor worked.

### 4.2. The Effects of Microstructures on Flexible Pressure Sensors’ Response Time

**Figure 8:**
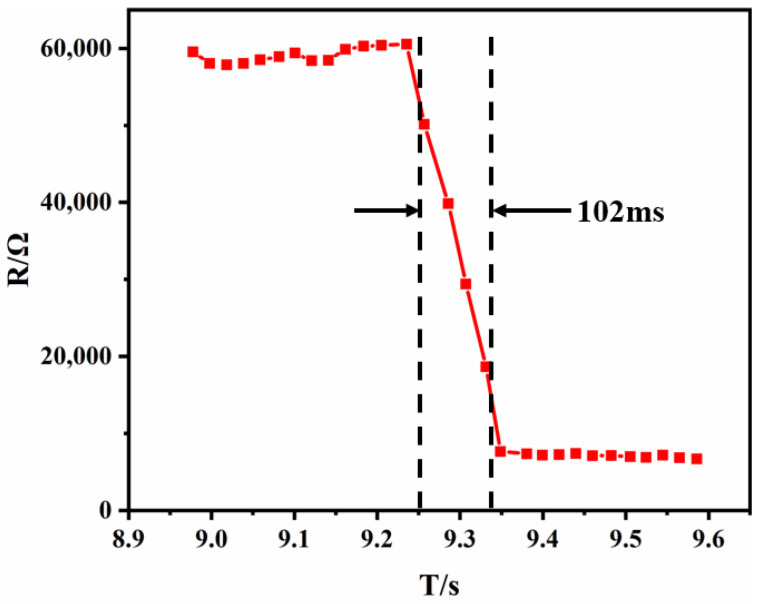
The curve of the sensor’s response time.

To further explore the response characteristics of the flexible pressure sensor with microstructures, the response time and repeatability of the sensor were studied. The results of the response time of sensor when loaded with pressure are shown in Figure 8. The test demonstrated that the response time was 112 ms, which was better than the sensor fabricated by casting [17].

### 4.3. The Stability of Flexible Pressure Sensors

**Figure 9:**
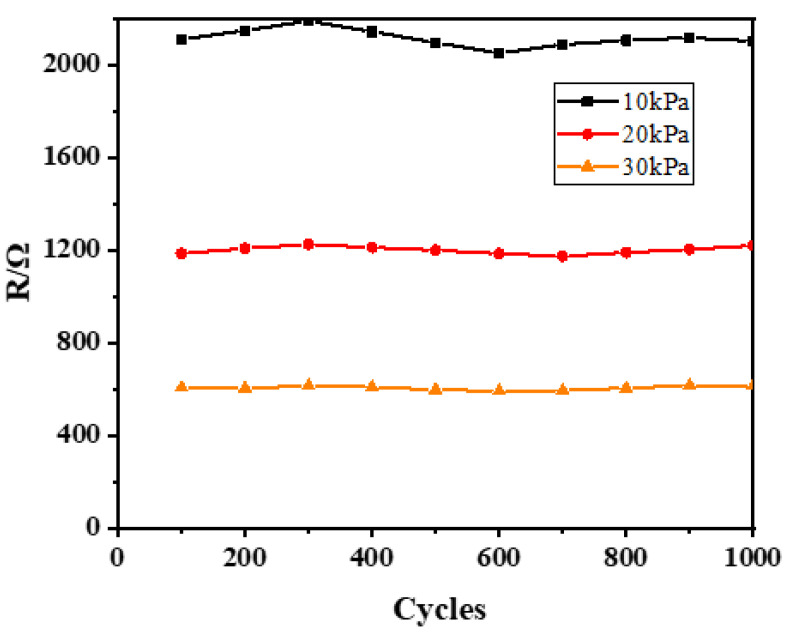
Results of the sensor repeatability test.

To test the stability of the sensor, the sensor was tested for 100 cycles under pressures of 10 kPa, 20 kPa, and 30 kPa, as displayed in Figure 9. The results showed that the relative resistance change rate of the flexible pressure sensors was almost unchanged and became steady. Therefore, the flexible pressure sensors fabricated by low-frequency ultrasonic resonance printing had good stability.

### 4.4. Economic Feasibility of Sensor Category Expansion

According to the results of printing the three-dimensional circular truncated cone microstructure described above, low-frequency ultrasonic resonance printing is an economical and convenient method to fabricate three-dimensional microstructures, and thus obtaining 3D microstructures could greatly simplify the fabrication process of flexible sensors, reducing the fabrication costs. However, it could print microstructures of various shapes only through the microstructure design software which came with the low-frequency ultrasonic resonance printing device. Therefore, low-frequency ultrasonic resonance printing technology could be applied for the fabrication of various flexible sensor devices, promoting the development of flexible electronic technology.

## 5. Conclusions

In this work, a low-frequency ultrasonic resonance printing technique was proposed to print the microstructures, and a flexible pressure sensor with microstructured electrodes was fabricated. Microstructured thin films were prepared on sandpaper by spin-coating based on CNT/PDMS composites, and the microstructures and the electrode were printed by low-frequency ultrasonic resonance printing. The performance of the flexible pressure sensor with and without microstructures was tested. It was demonstrated that the performance of the flexible pressure sensor with microstructures improved effectively. What is more, the flexible pressure sensor with microstructures could withstand a wide range of pressure from 0 to 30 kPa, and it had a fast response time and higher sensitivity (lower than 112 ms and 0.013 kPa^−1^, respectively).

## Data Availability

The data that support the findings of this study have not been made available but can be obtained from the author upon request.
